# Protein Aggregation and Protein Instability Govern Familial Amyotrophic Lateral Sclerosis Patient Survival

**DOI:** 10.1371/journal.pbio.0060170

**Published:** 2008-07-29

**Authors:** Qi Wang, Joshua L Johnson, Nathalie Y.R Agar, Jeffrey N Agar

**Affiliations:** 1 Department of Chemistry, Brandeis University, Waltham, Massachusetts, United States of America; 2 Volen Center, Brandeis University, Waltham, Massachusetts, United States of America; 3 Department of Biochemistry, Brandeis University, Waltham, Massachusetts, United States of America; 4 Department of Neurosurgery, Brigham and Women's Hospital, Harvard Medical School, Boston, Massachusetts, United States of America; University of California, San Francisco, United States of America

## Abstract

The nature of the “toxic gain of function” that results from amyotrophic lateral sclerosis (ALS)-, Parkinson-, and Alzheimer-related mutations is a matter of debate. As a result no adequate model of any neurodegenerative disease etiology exists. We demonstrate that two synergistic properties, namely, increased protein aggregation propensity (increased likelihood that an unfolded protein will aggregate) and decreased protein stability (increased likelihood that a protein will unfold), are central to ALS etiology. Taken together these properties account for 69% of the variability in mutant Cu/Zn-superoxide-dismutase-linked familial ALS patient survival times. Aggregation is a concentration-dependent process, and spinal cord motor neurons have higher concentrations of Cu/Zn-superoxide dismutase than the surrounding cells. Protein aggregation therefore is expected to contribute to the selective vulnerability of motor neurons in familial ALS.

## Author Summary

Amyotrophic lateral sclerosis (ALS), also known in America as Lou Gehrig's disease, is a fatal neurodegenerative disease with no effective treatment. Paralysis occurs as the result of the death of cells that connect the brain to various muscles, namely, the motor neurons of the brain and spinal cord. Ninety percent of ALS is sporadic and of unknown cause. A landmark discovery in ALS research was that mutations in the gene coding for Cu/Zn-superoxide dismutase cause at least 2% of ALS, and researchers have since discovered at least 119 such mutations. Neurologists also discovered that different mutations have remarkably different prognoses. For example, patients with the A4V mutation survive an average of 1 year after diagnosis, whereas patients with the H46R mutation survive an average of 18 years. Biochemists discovered that different mutations result in remarkably different physical properties, for example, stability of Cu/Zn-superoxide dismutase. In this article we apply an algorithm that predicts how fast a given Cu/Zn-superoxide dismutase will aggregate (stick to other proteins) and demonstrate that faster aggregation relates to faster death of ALS patients. We also demonstrate that loss of Cu/Zn-superoxide dismutase stability relates to faster ALS patient death. Our findings imply that aggregation of unfolded SOD1 is toxic for ALS patients, and in fact accounts for 69% of the variability in mutant Cu/Zn-superoxide-dismutase-linked familial ALS patient survival times.

## Abstract 3

Increased protein aggregation and decreased protein stability are shown to be central to familial amyotrophic lateral sclerosis etiology.

## Introduction

Amyotrophic lateral sclerosis (ALS) is an adult-onset neurodegenerative disease with roughly 10% of the cases being inherited or familial [1]. The cause of sporadic ALS (sALS) is unknown while familial ALS (fALS) is known to be caused by mutations in six different genes and six different chromosomal loci [2–4]. One of these genes encoding Cu/Zn-superoxide dismutase (SOD1) was found to associate with 20% of fALS, and at least 119 fALS-associated SOD1 mutations have been characterized in humans [1,5].

Fifteen years after the discovery that SOD1 mutations can cause ALS [1], the mechanisms of toxicity are still not well understood. The dominant inheritance of most SOD1 mutations and the literature as a whole indicate that SOD1 mutations result in a toxic gain of function rather than a loss of function [6–9]. Numerous hypotheses have been proposed, reviewed in [10,11], and can be broken down conceptually into the (nonexclusive) toxic mechanisms that converge to SOD1 protein structure–function and those that converge elsewhere (downstream effects). Popular hypotheses for SOD1 variant structure and function changes include decreased stability of apo or metallated SOD1 [12–15], increased hydrophobicity [16] and aggregation propensity [17,18], susceptibility to posttranslational modification [19–25], loss of metals [22,26–32], and aberrant chemistry [33–37]. Popular hypotheses for downstream effects [38–40] include impairment of axonal transport [41–43], impairment of proteasome [39,44,45] or chaperone activity [46,47], and mitochondrial [9,48–53] or endoplasmic reticulum–Golgi dysfunction [54,55]. Notably, the only potentially toxic property thus far shared by all fALS SOD1 variants is an increased propensity to form proteinaceous aggregates [56–62]. Due to the clinical similarities between fALS and sALS, research into SOD1 mutation-related fALS may provide insight into sporadic cases. Here we demonstrate that two properties, namely, increased aggregation propensity and instability (loss of stability), are major contributors to SOD1 toxicity in ALS patients. On the basis of these results we rationalize the determinants of aggregation, the selective vulnerability of neurons, and patient survival times.

## Results

### SOD1 Mutations Have Inherently Different Toxicities

**Figure 1:**
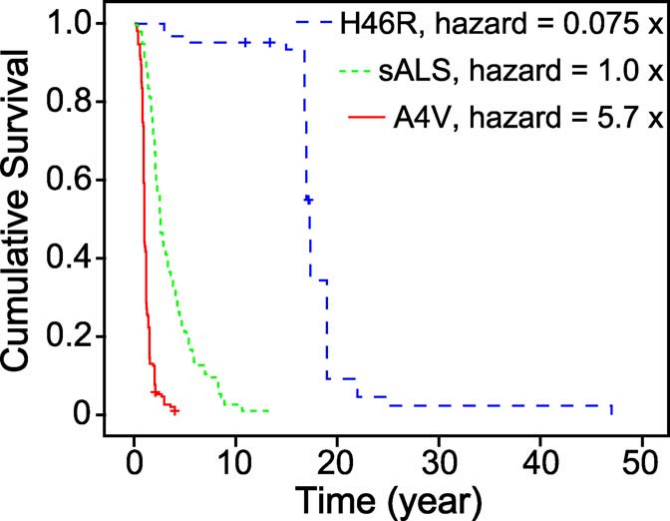
fALS Patients' Kaplan–Meier Survival Curves Illustrating Low and High Risk SOD1 Mutations Kaplan–Meier survival curves from patients with A4V (red) and H46R (blue) SOD1 mutations and sALS (green) are as shown. Disease durations from 205 patients with A4V SOD1 mutation, 63 patients with H46R SOD1 mutation, and 269 patients with sALS were used to generate these Kaplan–Meier survival curves.

The goal of this study is to discover the mechanisms of toxicity of fALS SOD1 mutations. Neurologists often publish the age at onset and the time from disease onset to death (also termed survival or disease duration) for their ALS patients, thereby enabling epidemiological studies that assess the risk of a given variable [63–66], which for this study include given mutations' relative toxicity and physical characteristics (physicochemical parameters). Previous studies revealed that different SOD1 mutations have inherently different toxicities (encode different mean disease durations) [67]. We expanded upon these studies with a larger set of fALS-causing SOD1 mutations as well as larger patient cohorts. Hazard ratios (relative risk of dying at a given time) of fALS SOD1 mutations and non-SOD1-related fALS compared to that of sALS were obtained from the Cox proportional hazard model (Table 1). From this result, fALS SOD1 mutations with data from at least five individual patients (full criteria for inclusion are defined in the Materials and Methods section) were significantly related to different hazards. Kaplan–Meier survival curves from patients with fALS-causing SOD1 mutations, non-SOD1-related fALS, and sALS were generated. Figure 1 illustrates that fALS SOD1 mutations encode different prognoses, ranging from considerably better (e.g., H46R, hazard rate = 0.075 × sALS) to considerably worse (e.g., A4V, hazard rate = 5.7 × sALS) than sALS. Moreover, the Log rank, Breslow, and Tarone–Ware tests, which also compare patient survival rates (i.e., each mutation versus every other fALS-causing SOD1 mutation, non-SOD1-related fALS, and sALS) using different mathematical functions, confirm that different SOD1 mutations have inherently different prognoses (Table 2).

**Table 1:**
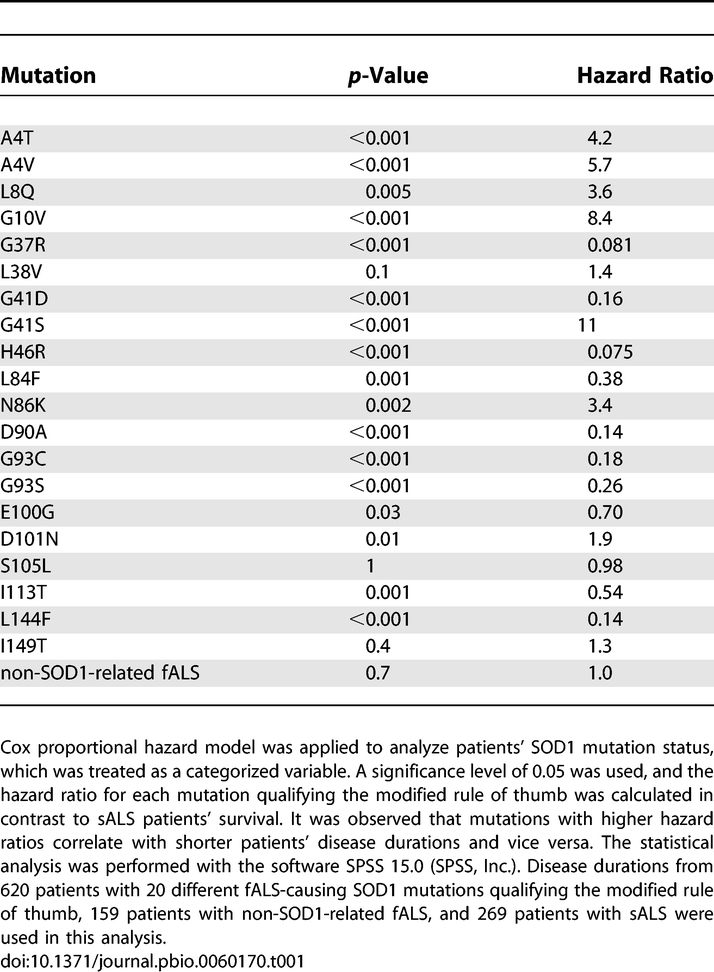
SOD1 Mutations Encode Inherently Different Prognoses

**Table 2:**
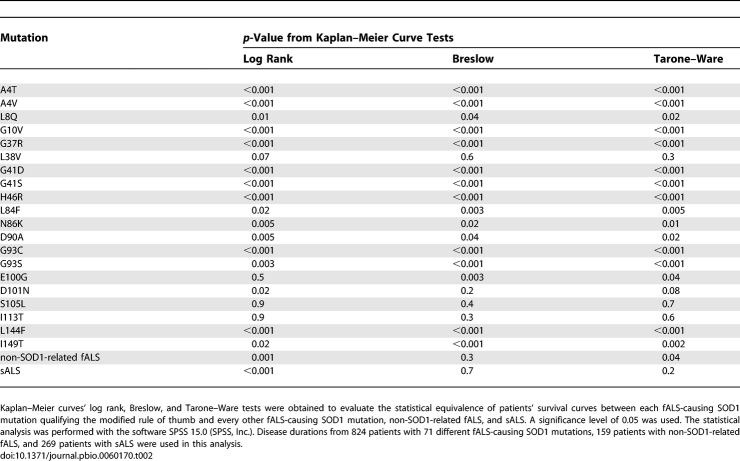
SOD1 Mutations Are Inherently Related to ALS Patients' Disease Duration

### SOD1 Variants' Gain of Hydrophobicity, Loss of α-Helix, and Gain of β-Sheet Propensity Are fALS Risk Factors, While Loss of Net Charge Is Protective

To test the hypothesis that changes to the physicochemical properties of SOD1 variants are toxic, specifically those properties known to influence protein aggregation, physicochemical properties for each protein variant (hydrophobicity, propensity to lose α-helices, form β-sheets, protein net charge, etc.) were evaluated in a Cox proportional hazard model (Table 3). The hazard ratios were significantly higher than 1.0 for mutations that either increase hydrophobicity, lose α-helices, or form β-sheets. In contrast, mutations that decrease the magnitude of the protein net charge correlate with hazard ratios significantly smaller than 1.0. These results indicate that changes in the SOD1 variants' properties, specifically increases in hydrophobicity and propensity to lose α-helices and to form β-sheets, correlate with decreased fALS patient survival, while decreases of the magnitude of net charge correlate with increased fALS patient survival, in contradiction with previous reports [15,68].

**Table 3:**
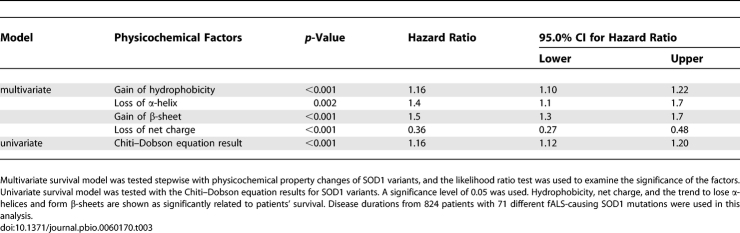
SOD1 Variants' Gain of Hydrophobicity, Loss of α-Helix, and Gain of β-Sheet Propensity Are ALS Risk Factors, While Loss of Net Charge Is Protective

Dobson and co-workers [69] introduced an equation (termed the Chiti–Dobson equation herein) to predict the changes of aggregation rates of unfolded peptides or proteins upon point mutations by their physicochemical properties. This equation was derived empirically by modeling how three physicochemical properties, hydrophobicity, secondary structure (including loss of α-helix and gain of β-sheet), and protein net charge, change upon mutations (the hazard analyses for each of these properties were reported in the previous paragraph). These physicochemical property changes then were related to changes in protein aggregation rate, yielding an equation that predicts how any mutation will change the rate of protein aggregation (the predicted change of aggregation rate is referred to as the aggregation propensity). Although this equation is empirical, it is based upon first physical/chemical principles and approximates how a given mutation will change the energy (and thus the equilibrium) between a solvated and an aggregated protein. The Chiti–Dobson equation is ln(ν_mut_/ν_wt_) = 0.633ΔHydr + 0.198(ΔΔ*G*_coil-α_ + ΔΔ*G*_β-coil_) – 0.491Δcharge, in which ln(ν_mut_/ν_wt_) represents change of aggregation rate upon mutation, and ΔHydr, ΔΔ*G*_coil-α_, ΔΔ*G*_β-coil_, and Δcharge represent the changes of hydrophobicity, free energy change for the process from α-helix to random coil, free energy change for the process from random coil to β-sheet, and protein net charge from the mutation, respectively. In their landmark study, it was demonstrated that increases in hydrophobicity, losses of α-helices, gains of β-sheets, and decreases in the magnitude of protein net charge increase the rate of protein aggregation.

### Protein Aggregation Propensity Is a Risk Factor of fALS

**Figure 2:**
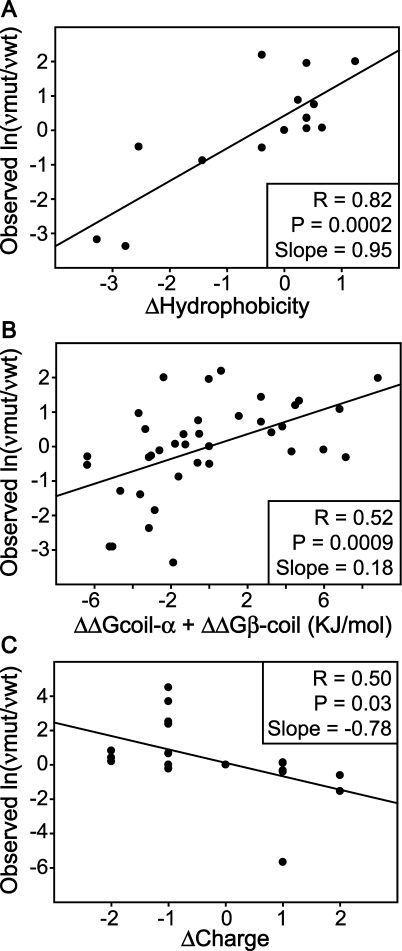
Rederivation and Validation of the Chiti–Dobson Equation The original Chiti–Dobson equation was rederived by correlating all known empirically measured mutation-related changes in protein aggregation rates as of 2005 with the corresponding changes in the physicochemical properties of charge, hydrophobicity, and secondary structure. Importantly, aggregation rate data were not taken directly from the publication that presented the Chiti–Dobson equation; instead these values were calculated from their respective original publication if applicable (Table 4). The dependence of observed ln(ν_mut_/ν_wt_) on hydrophobicity, secondary structure, and charge changes were still observed after the addition of extra protein aggregation data. (A) The relationship between observed ln(ν_mut_/ν_wt_) and Δhydrophobicity. To insure that the effect of hydrophobicity change was considered independent of other physiochemical properties, only mutations that had a Δcharge of 0 and a |ΔΔ*G*_coil-α_ + ΔΔ*G*_β-coil_| of less than 2.5 kJ/mol were considered. (B) The relationship between observed ln(ν_mut_/ν_wt_) and ΔΔ*G*_coil-α_ + ΔΔ*G*_β-coil_. To insure that the effect of secondary structure change was considered independent of other physiochemical properties, only mutations which had a Δcharge of 0 and a |Δhydrophobicity| of less than 3 kcal/mol were considered. (C) The relationship between the observed ln(ν_mut_/ν_wt_) and Δcharge. To ensure that the effect of charge change was considered independent of other physiochemical properties, only mutations that had a |Δhydrophobicity| of less than 3 kcal/mol and a |ΔΔ*G*_coil-α_ + ΔΔ*G*_β-coil_| of less than 2.5 kJ/mol were considered. Wild-type protein was used as a data point at (0,0) in all of the three graphs. The rederived slopes from this figure for the three factors, 0.95 for hydrophobicity, 0.18 for secondary structure, and −0.78 for charge, were applied to calculate aggregation propensities of fALS-causing SOD1 variants presented in Figure 4. Patient survival times were plotted against these aggregation propensities; the corresponding slope and *R* values differ less than 5% compared to the results in Figure 4 (unpublished data), validating the Chiti–Dobson equation.

The Chiti–Dobson equation and the many equations it inspired are robust and versatile, having successfully predicted aggregation rates of diverse disease-associated proteins [70], including amyloid β-peptide [69], tau [69], α-synuclein [69], amylin [69], lysozyme [71], etc. Moreover, increases in the predicted rates of aggregation of various mutations in amyloid β-peptide were shown to relate to increased neuronal dysfunction and degeneration in a *Drosophila* model of Alzheimer's disease [72]. To test the hypothesis that protein aggregation propensity is related to fALS patient survival, the Chiti–Dobson equation was used to predict the aggregation propensities of fALS-causing SOD1 mutations. We started this study by validating the Chiti–Dobson equation, taking all experimental protein aggregation rate data available at the inception of our study (data reported as of 2005, listed in Table 4) and recalibrating the equation. The detailed results of the validation are reported in Figure 2. In summary, the Chiti–Dobson equation was verified for use in fALS, and the statistical correlation between the physicochemical parameters (hydrophobicity, net charge, and secondary structure) and the aggregation propensity remained and changed only marginally. Since the time we validated the Chiti–Dobson equation, a number of papers also validated their general approach [73–75]. Even so, we have included our own analysis since it provides exposure to the physical basis of aggregation propensity. Furthermore, inclusion of this data makes this study self-contained so that all of the data necessary to support or disprove our model are contained herein. Notably, this paper's conclusions were the same using both the original and the recalibrated Chiti–Dobson equation.

**Table 4:**
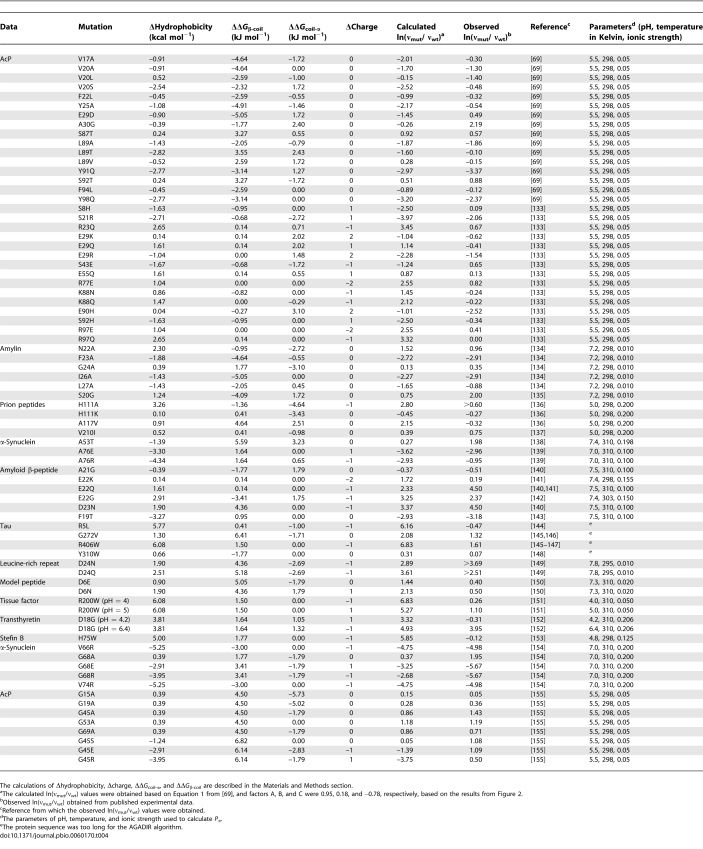
Data Used To Rederive the Chiti–Dobson Equation

**Figure 3:**
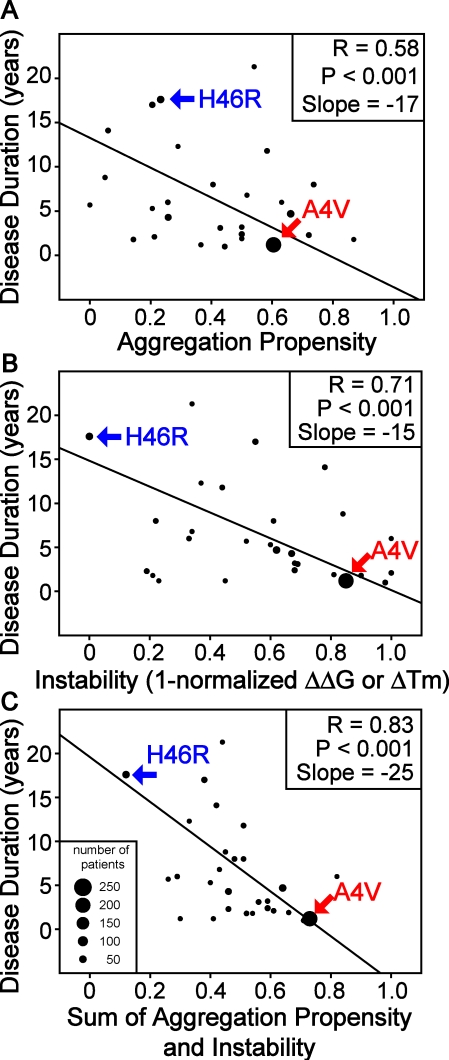
Synergistic Increases in SOD1 Aggregation Propensity and Increases of Instability Are Associated with Decreases in Survival for fALS Patients (Data Weighted by the Number of Patients) (A) An increase in aggregation propensity is associated with a decrease in fALS patient survival. Single point mutations of SOD1 found in fALS patients with corresponding reported stability values in (B) were considered. Aggregation propensities for each fALS mutation were calculated using the Chiti–Dobson equation, normalized such that 0 represents the least and 1 represents the most aggregation prone proteins, and the corresponding disease duration (survival) was plotted versus this value. (B) An increase in SOD1 instability is associated with severe disease. All instability values of apo SOD1 reported in the literature were normalized such that 0 represents the most and 1 represents the least stable proteins, and corresponding disease durations were plotted versus these values. (C) Increase in SOD1 aggregation propensity and gain of instability synergistically decrease patient survival. Normalized aggregation propensity in (A) and instability in (B) were summed and normalized to the range from 0 to 1, and patient survival was plotted versus this value. The data used in these three graphs (disease durations from 580 patients with 28 different fALS-causing SOD1 mutations with reported stability values) were weighted based on the number of patients for each mutation using SPSS version 15.0. Note that the correlation between the size of each data point and the number of patients for (A–C) is shown as an inset in (C).

**Figure 4:**
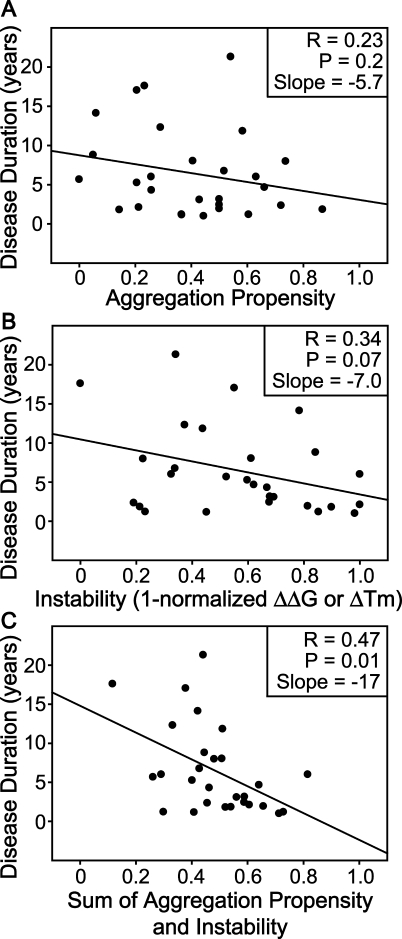
Synergistic Increases in SOD1 Aggregation Propensity and Losses of Thermodynamic Stability Are Associated with Decreased Survival of fALS Patients (Using Unweighted Data) (A) An increase in aggregation propensity is associated with decreased fALS patient survival. Single point mutations found in fALS patients with corresponding reported thermodynamic stabilities in (B) were considered. (B) A loss of SOD1 thermodynamic stability is associated with severe disease. (C) Increase in SOD1 aggregation propensity and gain of instability synergistically decrease patient survival. The data used in these three graphs (disease durations from 580 patients with 28 different fALS-causing SOD1 mutations with reported stability values) were treated equally regardless of the number of patients using the software SigmaPlot 9.0 (Systat Software, Inc.). SPSS 15.0 also was used on this analysis, and identical results were obtained.

The average patient survival times for different SOD1 variants with measured thermodynamic stabilities were plotted against corresponding predicted aggregation propensities, and linear regression analysis weighted by the number of patients for each mutation yielded *R* (multiple correlation coefficient, with a larger value indicating a stronger relationship) and *P* (value less than 0.05 implies a significant result) values of 0.58 and <0.001, respectively (Figure 3A). The severity of fALS thus is related to mutation-induced increases in SOD1 aggregation propensity. The same plot was performed with the linear regression analysis not weighted by the number of patients (Figure 4A), yielding *R* and *P* values of 0.23 and 0.2, respectively. Unfortunately, the published epidemiology data do not provide the information necessary to stratify for known ALS covariates, including lifestyle (diet and smoking) [76–79], palliative care [80], bulbar onset, etc., and weighted data are more likely to account for differences in these factors. The Chiti–Dobson equation results for all fALS-causing SOD1 mutations with patients' survival data also were evaluated in a univariate Cox proportional hazard model (Table 3). The hazard ratio for the Chiti–Dobson equation result was significantly higher than 1.0, which also indicates that aggregation propensity is a risk factor for fALS. Previous studies of Huntington's disease revealed an inverse relationship between the length of glutamine repeat of huntingtin and age of disease onset. The authors of this previous study concluded that disease onset correlates with rate of nucleation of aggregation [81]. We demonstrate here an inverse relationship between the rate of aggregation elongation *after nucleation* and the disease duration *after onset*.

### Protein Instability Is a Risk Factor for fALS

**Figure 5:**
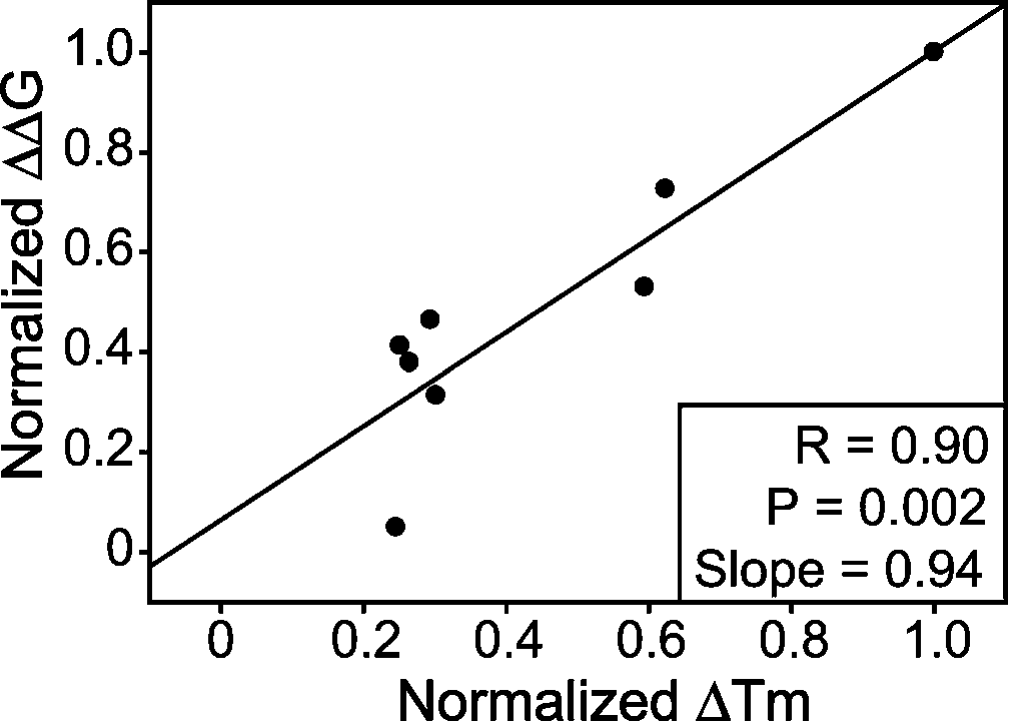
Correlation between Measures of Stability, Specifically Normalized Δ*T*_m_ and ΔΔ*G*, Reported from Different Groups for Apo SOD1 Variants The variants with both measured Δ*T*_m_ and ΔΔ*G* were plotted, and a good correlation between normalized Δ*T*_m_ and normalized ΔΔG was observed. This correlation provides the rationale for averaging Δ*T*_m_ and ΔΔ*G* and taking the average value as an indicator for stability of fALS-related variants. The normalized Δ*T*_m_ and ΔΔ*G* were obtained as described in the Materials and Methods section.

On the basis of our observation that predicted increased protein aggregation correlates with increased disease severity and previous data indicating that protein unfolding or misfolding promote aggregation [82–85], we tested the hypothesis that a loss of protein stability also could be a risk factor for ALS. For the sake of simplicity, we use the term instability throughout this article, with instability defined as the inverse of either the normalized ΔΔ*G* (unfolding free energy change difference between mutant and wild-type SOD1) or normalized Δ*T*_m_ (melting point difference between mutant and wild-type SOD1). Instability was considered for two reasons: (1) the Chiti–Dobson equation predicts the aggregation rates of unfolded proteins (it was derived from the aggregation rates of proteins in high trifluoroethanol concentrations that contained secondary but no tertiary structure), and therefore, formally, unfolding must occur prior to aggregation, and (2) unfolding is known to speed protein aggregation in vitro to the extent that without chemically induced unfolding induction periods extend from months to years, as demonstrated for SOD1 [32]. Aggregation in vivo therefore may require protein unfolding. Before using stability data published by different laboratories using different methods (melting point, which yields Δ*T*_m_, or chaotroph-induced unfolding, which yields ΔΔ*G*), we sought to determine the reliability of the data. If different laboratories reported similar values of stability for the same mutants, then the data could be deemed reliable. Therefore, all published measurements of apo SOD1 stability (metallated SOD1 calorimetry data often bear the characteristics of irreversible denaturation, probably via Cu-catalyzed disulfide bond formation, and is therefore less reliable) [15,31,60,86–88] were compiled, and the experimental values of ΔΔ*G* and Δ*T*_m_ were normalized to the range from 0 to 1 (described in the Materials and Methods section), with 0 representing the least stable, and 1 representing the most stable (highest stability) variant. Through the use of all of the data from mutants where ΔΔ*G* and Δ*T*_m_ were measured by different laboratories, a plot of normalized ΔΔ*G* versus normalized Δ*T*_m_ was created. Good interlaboratory correlation of measured stability values was observed (slope = 0.94, *R* = 0.90, *P* = 0.002; Figure 5), and we therefore deemed the stability data reliable for use.

Next, patient survival data for fALS-causing SOD1 variants were plotted against corresponding instability values, and linear regression analysis weighted by the number of patients for each mutation yielded *R* and *P* values of 0.71 and <0.001, respectively (Figure 3B). The same plot was performed with the linear regression analysis not weighted by the number of patients (Figure 4B), yielding *R* = 0.34 and *P* = 0.07. A gain of SOD1 instability (loss of stability) upon mutation therefore is related to decreased fALS patient survival. Increased in vitro instability is consistent with previous findings that the in vivo half-lives of SOD1 variants are decreased [89].

### Protein Aggregation Propensity and Protein Instability Are Synergistic Risk Factors for fALS

Previous results from computer simulations indicate a multistep process for aggregation via destabilization [90], encouraging us to understand the combined effect of aggregation propensity and protein instability upon ALS patient survival. On the basis of their respective multiple correlation coefficients and slopes, aggregation propensity and instability are equal contributors to fALS patients' survival. Moreover, no obvious correlation between protein instability and aggregation propensity was observed for the SOD1 variants used in Figure 3 (Figure S1), indicating that increased instability is not responsible for the increased predicted protein aggregation propensity. The combination of instability and aggregation propensity represents the relative energy in proceeding from folded to unfolded apo SOD1 and then from unfolded to aggregated states. Patient survival was plotted against corresponding summed instability and aggregation propensity values. A linear regression analysis weighted by the number of patients for each mutation yielded *R* and *P* values of 0.83 and <0.001, respectively (Figure 3C). The same plot was performed with the linear regression analysis not weighted by the number of patients (Figure 4C), yielding *R* = 0.47 and *P* = 0.01. The improved statistical result of predicting patient survival after combining instability and aggregation propensity indicates that aggregation occurs from unfolded or partially unfolded SOD1. The stability data used herein were for apo SOD1, and therefore the absence of metals is implicit. The *R* ^2^ value was 0.69 from the weighted data, indicating that 69% of the intrinsic variability these fALS patients' survival resulted from the combination of increased aggregation propensity and instability. Additionally, aggregation propensity and instability were evaluated in a Cox proportional hazard model (Table 5). The hazard ratios were significantly higher than 1.0 for both factors. The sum of aggregation propensity and instability also was evaluated in a univariate Cox proportional hazard model (Table 5). The hazard ratio for this sum was also significantly higher than 1.0, further indicating that aggregation propensity and instability are synergistic risk factors for fALS. Note that the aggregation propensity and instability tested in Table 5 were normalized to the range from 0 to 1 (as in Figures 3 and 4), while the values tested in Table 3 were not normalized. As a result of normalization, which decreased the value range of tested factors, the hazard ratios of Table 5 are much larger than Table 3, and therefore the large values of hazard ratios reported in Table 5 should not be overinterpreted. Significantly, a fALS patient with an SOD1 mutation of relatively low aggregation propensity and high stability is expected to survive longer after disease onset. It has not escaped our attention that the rate of protein aggregation has implications in both sporadic diseases and aging; for example, the toxicity of a given posttranslational modification is a function of its effect on protein stability and aggregation propensity.

**Table 5:**
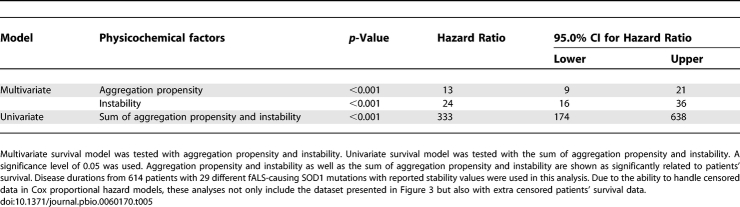
SOD1 Variants' Aggregation Propensity and Instability Are ALS Risk Factors

## Discussion

We describe here synergistic gains of toxic functions of SOD1 in ALS. These are the first results in any neurodegenerative disease demonstrating that protein instability and aggregation propensity are synergistic risk factors. The fact that there are two synergistic risk factors rather than a single toxic gain of function probably has delayed the discovery of the mechanisms of fALS mutant SOD1 toxicity. The SOD1 stability data used in this paper were measured from apo SOD1, and the aggregation rate data used to create the Chiti–Dobson model were from in vitro unfolded proteins. Therefore, formally, the combination of instability and aggregation propensity represents the relative energy in proceeding from apo folded to unfolded SOD1 and then from unfolded to aggregated states. It has been demonstrated experimentally that apo SOD1 has a faster rate of aggregation than that of holo forms [32]. Partial unfolding/misfolding also can lead to aggregation [28,91–96], and our results cannot rule out a role for the aggregation of partially folded, including metallated, SOD1. Previous studies revealed a correlation [15] and conversely a lack of correlation [86] between SOD1 variant stability and patient disease duration. Correlation between SOD1 variant stability and patient disease duration, however, required that the authors omit stability data of 4 of the 15 variants from their regression analysis (on the basis that these variants change the net charge of SOD1).

As presented in Table 3, SOD1 variants' loss of net charge correlates with increased patient survival, while gain of hydrophobicity, loss of α-helix, and gain of β-sheet propensity are ALS risk factors. On the basis of Dobson and co-workers' related work [69,73,97], a loss of net charge is predicted to increase the aggregation propensity of unfolded proteins. If aggregation is toxic, then one would expect loss of net charge to be toxic. In contrast to the synergistic effects for aggregation propensity and instability presented in Table 5, the correlation of loss of net charge with increased survival has an effect of decreasing the hazard ratio presented in the univariate model presented in Table 3. We demonstrate that mutations causing the entire protein to approach neutrality are protective in the context of fALS (Table 3) rather than deleterious as proposed by Oliveberg and co-workers [15,68]. These results should be cautiously interpreted since in contrast to our Cox proportional hazard model result that loss of net charge is protective, the mean patient survival for loss of net charge and gain of net charge mutations, unweighted by the number of patients, are 7.1 and 6.9 years, respectively. Further study clearly is required to understand the role of charge in ALS etiology.

In contrast with the strong familiality shown for disease duration after onset (Table 1), SOD1-mediated ALS showed modest familiality with respect to onset, accounting for only 42% of the variability in A4V and D90A fALS patients [98], and with only G37R and L38V mutations of SOD1 being significant covariates of age of onset [67]. The same analysis shown in Figures 3 and 4 was performed using age at disease onset rather than disease duration as the dependant variable (Figure S2), and little or no relationship between disease onset and aggregation propensity or instability was observed. The Chiti–Dobson equation predicts the rate of aggregation after nucleation (rate of elongation). It is tempting therefore to speculate that the rate of nucleation is a determinant of age at onset. Testing this hypothesis would require the development of a model that can predict nucleation rates based upon physicochemical parameters, a task that is hampered by the stochastic nature of in vitro nucleation times [99,100] but that should now be possible given our recent development of methods for modeling in vitro nucleation kinetics [101].

Although our model accounts for 69% of the variability in fALS patient survival after onset, there are clearly genetic components of fALS that our model cannot account for. For example, while D90A is normally a dominantly inherited mutation in North America, 2.5% of people in Sweden and Finland are heterozygous asymptomatic carriers of the D90A SOD1 mutation [102,103] and require two mutant alleles before presenting ALS symptoms. Notably, our results and conclusions were unaffected by including or excluding D90A survival times during data analysis.

It is postulated that diseases for which protein aggregation contributes to patient death will (1) develop in cells with the highest concentration of the aggregation-prone protein in accordance with the concentration dependence of aggregation rates [101,104] and (2) have a prognosis influenced by the aggregation propensity of the aggregating protein, in accordance with the results reported herein. Motor neurons are the cells in the ventral horn of the spinal cord with the highest SOD1 concentration [39,105], perhaps explaining an aspect of the selective vulnerability of these cells.

Protein aggregation is a hallmark of many neurodegenerative diseases, including ALS. The toxicity of aggregation is fiercely debated (reviewed in [106,107]), fueled by reproducible evidence that aggregates can be toxic [108,109], have no effect [110], or be protective [111]. We propose that aggregates on either extreme of size, i.e., small protofibrillar aggregates [108,112] or aggregates large enough to clog axons, are more toxic, while midsize microscopically visible aggregates are less toxic [106]. Our data indicate that the increased aggregation propensity of SOD1 is related to decreased survival of ALS patients. Notably aggregation of SOD1 has been demonstrated in fALS [113,114] and a subset of sALS patients [114,115], 18 fALS rodent models of 13 different SOD1 mutations [7–9,23,59,116–128], and at least 13 SOD1 mutants in cell models [56,58,59,116,118,119,129–131].

## Materials and Methods

### Familial ALS patients' disease duration and age of onset.

Familial ALS patients' data were taken from all of the available literature. Disease duration was initiated with onset of the first symptoms until the patient's death or when respiratory assistance was required for patients' survival. The average duration and onset for each mutation were calculated as the weighted average based on the number of patients (Table 6). If the patients were still reported to be alive without respiratory assistance at the end of a study, then their disease durations were not used to calculate the average unless the known duration value was larger than the average calculated with only durations from patients deceased or with respiratory assistance. For studies reporting average disease duration and Kaplan–Meier curves, the reported average durations were used to calculate the weighted averages. The current unavailability of http://www.alsod.org/ made it impossible to review the references provided by the website (from which we had taken survival times before it became unavailable), which created the risk of counting a patient's disease onset or survival twice, and made reproducing our study impossible for other groups. We therefore opted not to report data from this website in this study, thereby eliminating no more than 67 (there were 67 http://www.alsod.org/ patients' data without accompanying literature references that may, or may not, have been represented by our literature search) of 1319 patients' data. However, we did perform a complete, alternative set of analyses that did include http://www.alsod.org/ data (unpublished data), and the statistical correlations in the figures and tables shown herein persisted. Mean values of disease durations also were obtained from Kaplan–Meier curves and tested on SOD1 mutations with known experimental thermodynamic stabilities, and the results were comparable to those in Figure 3. Since the weighted average method can provide disease duration regardless of the number of patients, we opted for its use.

**Table 6:**
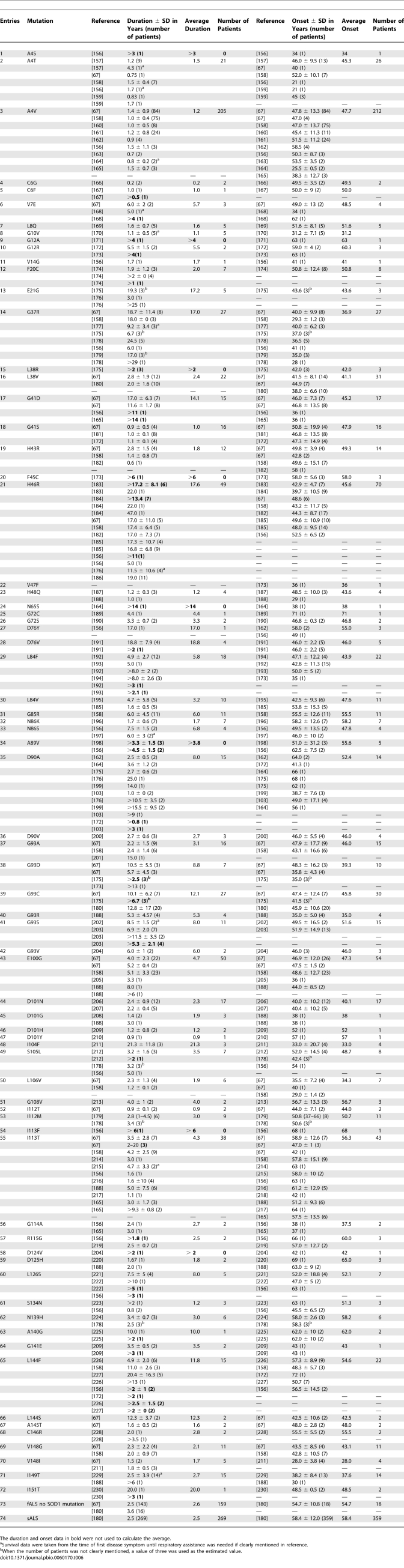
Disease Durations and Age of Onsets for SOD1 Mutation Related fALS, Non-SOD1-Related fALS, and sALS Patients

### Kaplan–Meier survival curves, the log rank tests, and the Cox proportional hazard model.

Kaplan–Meier curves of survival for different fALS-causing SOD1 mutations, non-SOD1-related fALS, and sALS were generated. The hazard ratios of different fALS-causing SOD1 mutations and non-SOD1-related fALS compared to sALS were tested as a category variable by Cox proportional hazard model analysis. For studies reporting Kaplan–Meier curves but without individual patients' data, the Engauge Digitizer 4.1 software was used to obtain coordinates for cumulative survival at each time point. This information was used to calculate the number of patients not surviving at each time point under the assumption that there is no censored patient (with unknown exact survival time because of being alive at the end of study, lost to follow-up, or withdrawal from the study) within the course of survival curves. For cumulative survival not reaching 0 at the end of study, those fractions of patients were treated as censored. The error of the estimated number of patients is less than 5% of the number reported. To eliminate the chance that one or two patients' survival data bias the analysis result, a rule of thumb [132] requiring that each tested fALS-related SOD1 mutation includes at least five noncensored patients was applied. Since patients' survival was reported only as an average from a group of patients and individual patient's survival information was not described in some publications, one or two publications' survival data might bias the analysis result. To eliminate this chance of bias, the rule of thumb was modified as requiring at least five independent descriptions of noncensored patients' survival data (a reported average without individual patient's survival information was treated as one description). The statistical analysis was performed with the software SPSS 15.0 (SPSS, Inc.).

### Aggregation propensity calculated from the Chiti–Dobson equation.

The hydrophobicity, β-sheet propensity, and charge values for the amino acid residues were obtained from the Supplementary Information of [69]. While applying the AGADIR algorithm at http://www.embl-heidelberg.de/Services/serrano/agadir/agadir-start.html to obtain α-helical propensities for wild-type (wt) and mutant (mut) *P*_α_^wt^ and *P*_α_^mut^ values for ΔΔ*G*_coil-α_ calculation for human SOD1, the parameters of pH 7, 310 K, and ionic strength of 0.100 were used. For the protein human SOD1, the N terminus is acetylated, and the C terminus is free in vivo. After the prediction at the residue level was output, the value in the column “Hel” at a specific residue was taken as *P*_α_. If a value of 0 for *P*_α_ was obtained, then 0.1 was added to both *P*_α_^wt^ and *P*_α_^mut^ values for the correct mathematical meaning of ln(*P*_α_^wt^/*P*_α_^mut^) (F. Chiti, personal communication). The Chiti–Dobson equation terms, Δhydrophobicity, Δcharge, ΔΔ*G*_coil-α_, and ΔΔ*G*_β-coil_, were calculated based on equations illustrated in the legend of Table 1 of [69]. The ln(ν_mut_/ν_wt_) values were calculated based on Equation 1 from [69] and normalized from 0 to 1 using the equation normalized aggregation propensity = (aggregation propensity before normalization – MIN_ap_)/(MAX_ap_ – MIN_ap_), with MIN_ap_ and MAX_ap_ as the minimum and maximum aggregation propensities of fALS-causing mutations with known thermodynamic stabilities, respectively, so that the larger normalized values correlate to larger aggregation propensities.

### Normalized ΔΔ*G*.

The free energy change difference (ΔΔ*G*) and melting temperature difference (Δ*T*_m_) of unfolding a pathogenic variant and wild-type protein are parameters used to characterize the thermodynamic stability of a protein. ΔΔ*G* values were taken from Table 2 of [15]. To graph with other protein stability data, the ΔΔ*G* values were normalized by applying the equation normalized ΔΔ*G* = (ΔΔ*G* values before normalization – MIN_ΔΔ*G*_)/(MAX_ΔΔ*G*_ – MIN_ΔΔ*G*_), with MIN_ΔΔ*G*_ and MAX_ΔΔ*G*_ as the minimum and maximum values of ΔΔ*G* in this dataset, respectively.

### Normalized Δ*T*_m_.

Δ*T*_m_ values were taken from Table 1 of [86], Table 1 of [60], Table 2 of [87], Table 3 of [88], and Table II of [31]. Δ*T*_m_ values from [60,87,88] were averaged for each mutation. Those results then were averaged with the Δ*T*_m_ values from [31,86] to determine the Δ*T*_m_ values for given mutations. The normalized Δ*T*_m_ values were obtained by applying the equation normalized Δ*T*_m_ = (average Δ*T*_m_ values before normalization – MIN_Δ*T*m_)/(MAX_Δ*T*m_ – MIN_Δ*T*m_), with MIN_Δ*T*m_ and MAX_Δ*T*m_ as the minimum and maximum values of averaged Δ*T*_m_ values in this dataset, respectively.

### Thermodynamic instability of SOD1 variants.

The instability values for SOD1 variants were obtained from the equation normalized instability = 1 – average of normalized ΔΔ*G* and normalized Δ*T*_m_ for each mutation, so instability values are simply (1 – normalized ΔΔ*G* or Δ*T*_m_), and larger values correlate to less stable variants.

The normalized aggregation propensity and instability for each variant were summed and normalized to the range from 0 to 1 to consider the two factors together.

## Supporting Information

Alternative Language Abstract S1 Translation of the Abstract into Chinese by Qi Wang (62 KB PDF)

Figure S1 Lack of Correlation between SOD1 Aggregation Propensity and SOD1 Instability The same dataset for fALS-associated SOD1 mutations shown in Figures 3 and 4 were considered. The predicted aggregation propensities and instabilities from 28 different fALS-causing SOD1 mutations were plotted using the software SigmaPlot 9.0 (Systat Software, Inc.).
(213 KB AI).

Figure S2 Little or No Correlation between SOD1 Aggregation Propensity, SOD1 Instability, or the Sum of Aggregation Propensity and Instability with fALS Patients' Age of Onset The relationship between SOD1 aggregation propensity (A, D), instability (B, E), or sum of aggregation propensity and instability (C, F) with fALS patients' age of onset are presented. The linear regressions presented in (A–C) were weighted by the number of patients for each mutation using SPSS version 15.0 (SPSS, Inc.). The correlation between the size of each data point and the number of patients for (A-C) is shown as an inset in (C). The linear regressions presented in (D–F) were treated equally regardless of the number of patients for each mutation (unweighted) using the software SigmaPlot 9.0 (Systat Software, Inc.). The age of onset data presented in these six graphs are from 649 patients with 29 different fALS-causing SOD1 mutations with reported stability values. Aggregation propensity, instability, and sum of aggregation propensity and instability were obtained as described in the Materials and Methods section. Aggregation propensity, instability, or sum of aggregation propensity and instability has little or no correlation with patients' age of onset.
(261 KB AI).

## Abbreviations

ALS: amyotrophic lateral sclerosis
fALS: familial ALS
mut: mutant
sALS: sporadic ALS
SOD1: Cu/Zn-superoxide dismutase
wt: wild-type
