# Supplementary material for: Protein Aggregation and Protein Instability Govern Familial Amyotrophic Lateral Sclerosis Patient Survival
Source: PLoS Biol. 2008 Jul 29;6(7):e170. doi: 10.1371/journal.pbio.0060170 (PMC2486295; doi:10.1371/journal.pbio.0060170)
Supplement: Alternative Language Abstract S1 — (62 KB PDF) [file pbio.0060170.sd001.pdf]

蛋白质聚集和蛋白质的不稳定性是决定家族型肌萎缩侧索硬化症病人生存的因素

摘要

同肌萎缩侧索硬化症(ALS)，帕金森氏病，和阿兹海默症相关的突变所引起的“获得的毒性功能”的本质是一个尚有争论的问题。目前还没有一种充分解释神经退化性疾病的模型。我们的研究结果显示，蛋白质增加的聚集倾向(打开的蛋白质将聚集的倾向)和蛋白质减少的稳定性(蛋白质将打开的倾向)，这两种因素的协同作用是 ALS 的重要的因素。这两种因素一起揭示了 69%的和铜/锌超氧化物歧化酶突变相关的家族型 ALS 病人发病后的生存时间。蛋白质的聚集是一个依赖于浓度的过程，并且同其周围的其它细胞相比，脊髓运动神经元的铜/锌超氧化物歧化酶的浓度更高。因此在家族型的 ALS 中，我们可以预计蛋白质聚集是造成运动神经元的选择性脆弱的原因。

作者总结

肌萎缩侧索硬化症(ALS)，在美国又称鲁盖瑞氏症，是一种目前没有有效的治疗方法的致命性的神经退化性疾病。连接大脑到各种肌肉的脑和脊髓的运动神经元细胞的死亡造成患者的瘫痪。百分之九十的 ALS 是分散型的未知起因的。迄今为止在 ALS 研究领域中的最影响深远的发现是由铜/锌超氧化物歧化酶的基因突变导致至少 2%的 ALS，从那以后的研究相继发现了至少 119 个相关的突变。神经学家也发现不同的突变和显著不同的预后相关。例如，有 A4V 突变的病人在诊断以后平均仅能生存一年，而有 H46R 突变的病人在诊断以后平均能生存 18 年。生化学家发现不同的突变导致显著不同的物理性质的变化，例如铜/锌超氧化物歧化酶的稳定性的变化。在本文中，我们运用了一种现有的算法来预测铜/锌超氧化物歧化酶发生各种突变后聚集的快慢，并且显示其更快速的聚集与 ALS 患者发病后更快速的死亡相关联。另外，铜/锌超氧化物歧化酶减少的稳定性也与 ALS 患者发病后更快速的死亡相关联。本文的结果显示出打开的铜/锌超氧化物歧化酶的聚集对于 ALS 患者是具有毒性的，并且 69%的由于铜/锌超氧化物歧化酶突变引起的家族型 ALS 患者发病后的生存时间由此决定。
