# Supplementary figures and images for: Protein Aggregation and Protein Instability Govern Familial Amyotrophic Lateral Sclerosis Patient Survival
Source: PLoS Biol. 2008 Jul 29;6(7):e170. doi: 10.1371/journal.pbio.0060170 (PMC2486295; doi:10.1371/journal.pbio.0060170)

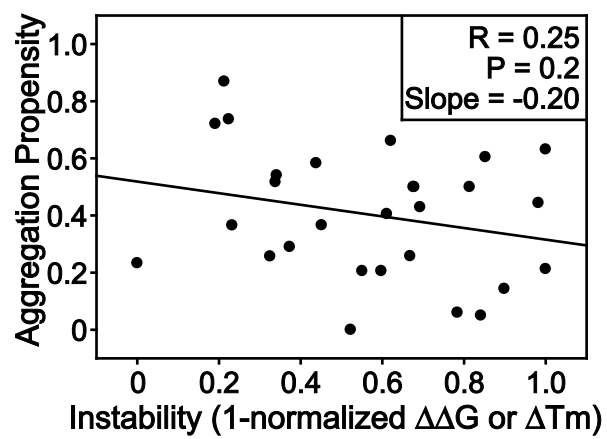

Supplement: Figure S1 — The same dataset for fALS-associated SOD1 mutations shown in Figures 3 and 4 were considered. The predicted aggregation propensities and instabilities from 28 different fALS-causing SOD1 mutations were plotted using the software SigmaPlot 9.0 (Systat Software, Inc.). (213 KB AI). [file pbio.0060170.sg001.pdf]

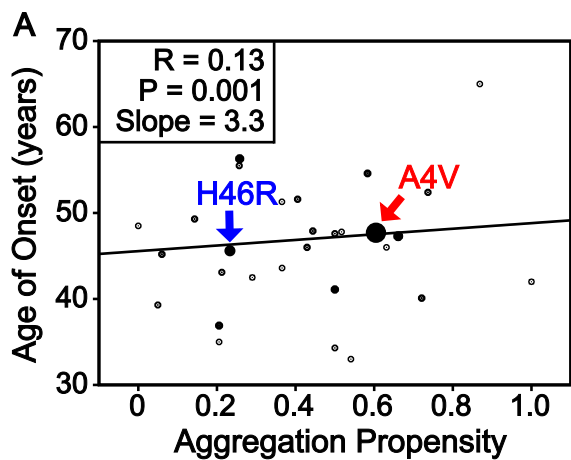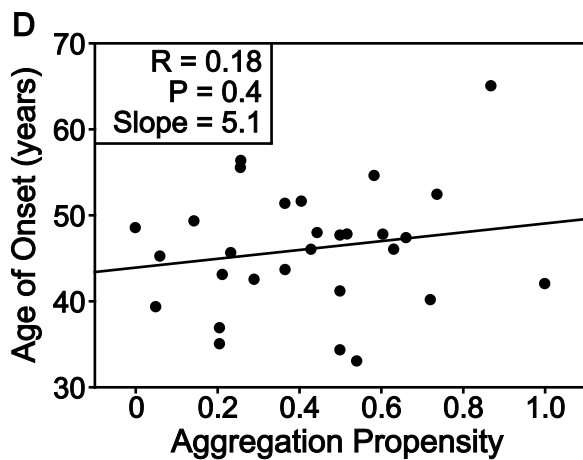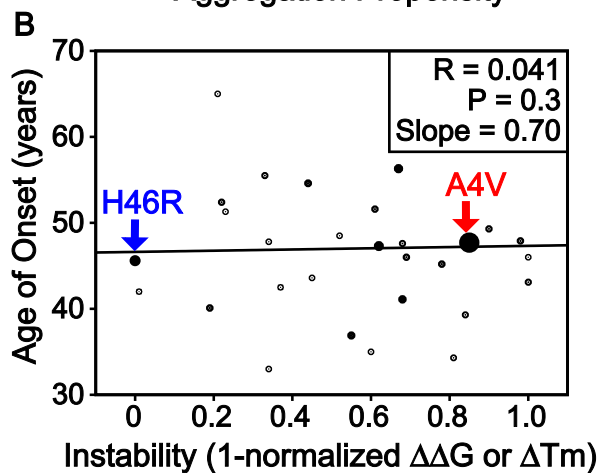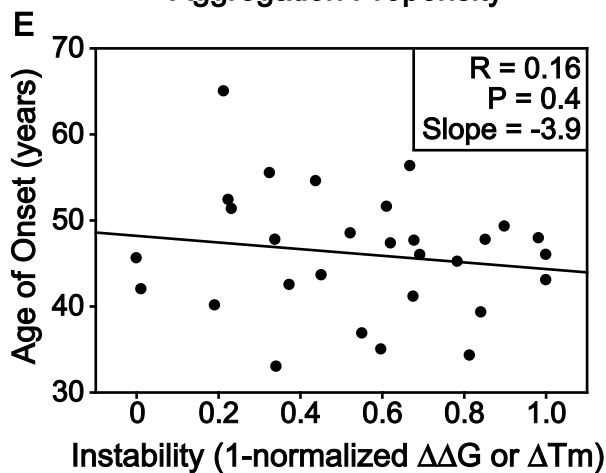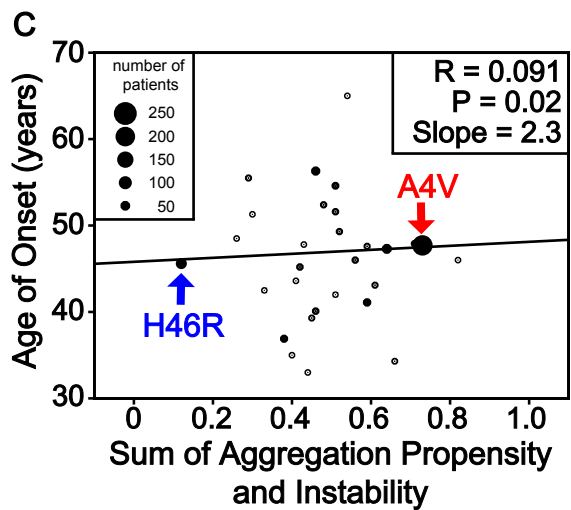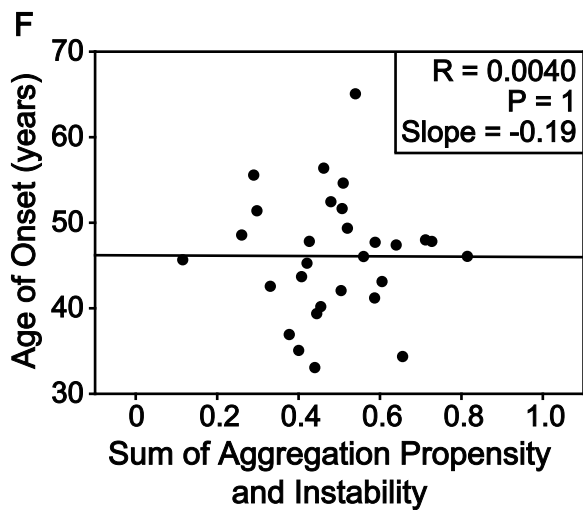

Supplement: Figure S2 — The relationship between SOD1 aggregation propensity (A, D), instability (B, E), or sum of aggregation propensity and instability (C, F) with fALS patients' age of onset are presented. The linear regressions presented in (A–C) were weighted by the number of patients for each mutation using SPSS version 15.0 (SPSS, Inc.). The correlation between the size of each data point and the number of patients for (A-C) is shown as an inset in (C). The linear regressions presented in (D–F) were treated equally regardless of the number of patients for each mutation (unweighted) using the software SigmaPlot 9.0 (Systat Software, Inc.). The age of onset data presented in these six graphs are from 649 patients with 29 different fALS-causing SOD1 mutations with reported stability values. Aggregation propensity, instability, and sum of aggregation propensity and instability were obtained as described in the Materials and Methods section. Aggregation propensity, instability, or sum of aggregation propensity and instability has little or no correlation with patients' age of onset. (261 KB AI). [file pbio.0060170.sg002.pdf]
